# Supplementary material for: Cell-fate transition and determination analysis of mouse male germ cells throughout development
Source: Nat Commun. 2021 Nov 25;12:6839. doi: 10.1038/s41467-021-27172-0 (PMC8617176; doi:10.1038/s41467-021-27172-0)
Supplement: Supplementary file 1 — Supplementary Information [file 41467_2021_27172_MOESM1_ESM.pdf]

Supplementary Information

Cell-fate transition and determination analysis of mouse male germ cells throughout development

Jiexiang Zhao, Ping Lu, Cong Wan, Yaping Huang, Manman Cui, Xinyan Yang, Yuqiong Hu, Yi Zheng, Ji Dong, Mei Wang, Shu Zhang, Zhaoting Liu, Shuhui Bian, Xiaoman Wang, Rui Wang, Shaofang Ren, Dazhuang Wang, Zhaokai Yao, Gang Chang, Fuchou Tang & Xiao-Yang Zhao

Correspondence should be addressed to G. C. (email: changgang@szu.edu.cn), F. T. (email: tangfuchou@pku.edu.cn) or to X. Z. (email: zhaoxiaoyang@smu.edu.cn).

Contents for Supporting Information

I. Supplementary Table

II. Supplementary Figures

Supplementary Table 1

Numbers of single cells collected (numbers of embryos or male testicular gonads used) per sampled time-point														
Time-point	E6.5	E7.5	E8.5	E9.5	E10.5	E11.5	E12.5	E13.5	E14.5	E15.5	E16.5	E17.5	E18.5	PND0
R1	480 (20)	480 (18)	48 (9)	96 (12)	96 (9)	144 (9)	96 (11)	144 (9)	144 (4)	144 (4)	144 (4)	96 (2)	96 (2)	192 (1)
R2	192 (16)	280 (16)	96 (13)	96 (14)	96 (10)	96 (8)	96 (10)	144 (11)	144 (4)	144 (4)	144 (4)	96 (2)	96 (2)	192 (1)
R3	96 (10)	96 (9)	48 (6)		120 (10)									
R4		48 (5)												
Total	768	904	192	192	312	240	192	288	288	288	288	192	192	384

Time-point	PND1	PND2	PND3	PND4	PND5	PND6	PND7	PND8	PND10	PND12	PND14	PND30	PND35	Adult
R1	192 (1)	128 (1)	192 (1)	192 (1)	192 (1)	128 (1)	192 (1)	192 (1)	192 (1)	96 (1)	480 (1)	576 (1)	384 (1)	384 (1)
R2	192 (1)	192 (1)	192 (1)	192 (1)	192 (1)	192 (1)	192 (1)	192 (1)	192 (1)	240 (1)	384 (1)	480 (1)	344 (1)	480 (1)
Total	384	320	384	384	384	320	384	384	384	336	864	1056	728	864

Total	11,896 (After QC: 11,598)

Supplementary Table 1. Sample information of the cells analyzed in this study.

Supplementary Fig. 1 related to Fig. 1

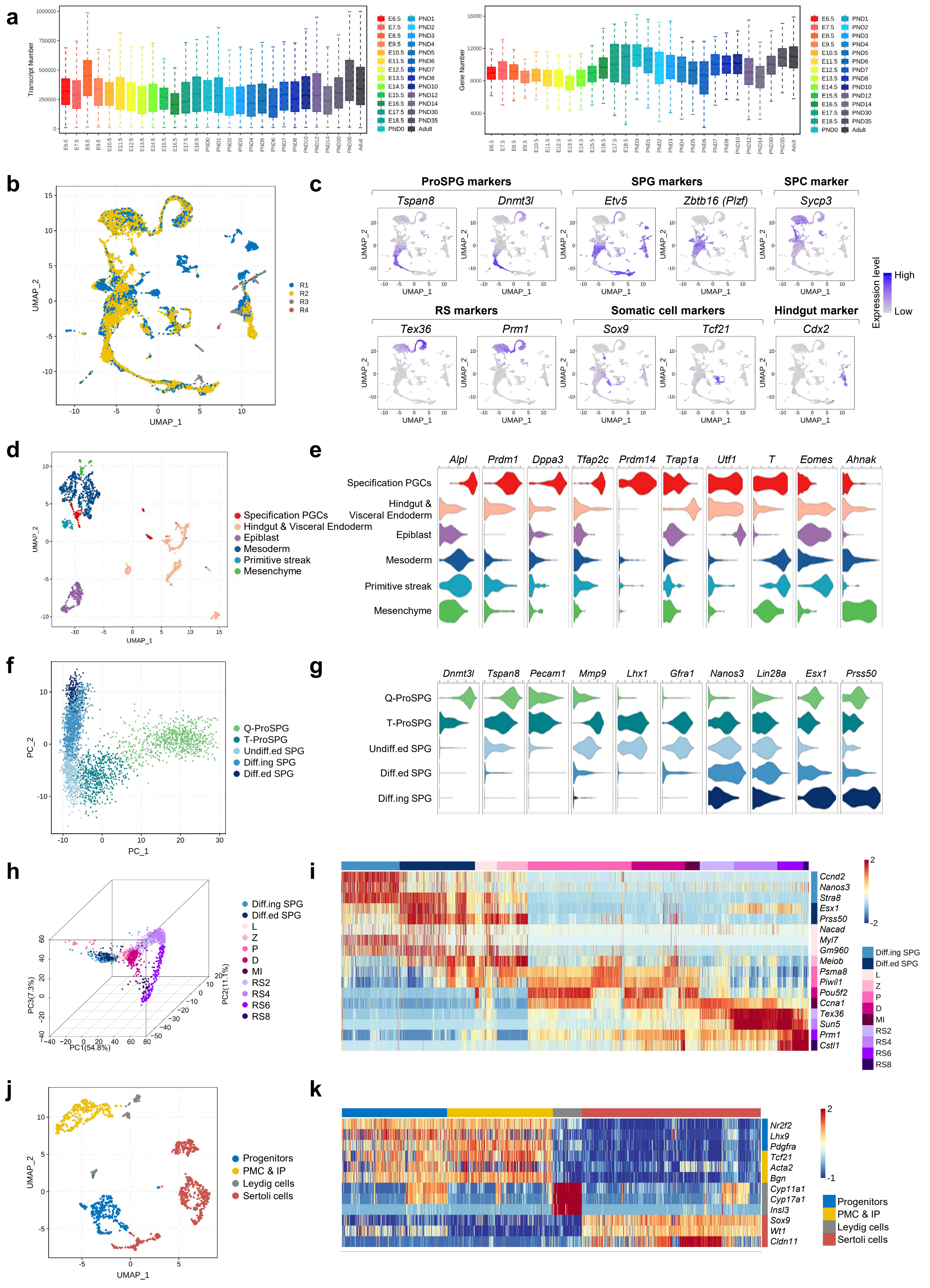

Supplementary Fig. 1 Identification of mouse male gonadal/testicular cells and quality control of scRNA-seq data.

a, Boxplot of the number of transcripts (left) and number of genes (right) detected in each single cell. Centre line, median; box limits, interquartile range (IQR); whiskers, minima and maxima within 1.5 * IQR. **b,** UMAP plots are colored by the biological replicates sampled. **c,** UMAP plots are colored by expression levels for selected marker genes. Aliases of some genes are also shown. The color key from gray to dark blue indicates low to high expression levels. **d,** Re-clustering of E6.5-8.5 mixed cells in cluster 1 by UMAP. Cells are colored by indicated cell types. **e,** Violin plots of selected marker genes of each cell types in cluster 1. **f,** Re-clustering of clusters 5 and 6 by PCA. Cells are colored by indicated cell types. **g,** Violin plots of selected marker genes of each cell type in clusters 5 and 6. **h,** Re-clustering analysis of clusters 7 and 8 by 3D PCA. Cells are colored by indicated cell types. L, leptotene; Z, zygotene; P, pachytene; D, diplotene; MI, metaphase I; RS, round spermatids. **i,** Heatmap of representative marker genes for SPG, SPC and RS. The color key from blue to red blue indicates low to high expression levels. L, leptotene; Z, zygotene; P, pachytene; D, diplotene; MI, metaphase I; RS, round spermatids. **j,** Re-clustering of clusters 9 and 10 by UMAP. Cells are colored by indicated cell types. PMC & IP, peritubular myoid cells & interstitial progenitors. **k,** Heatmap of marker genes of four somatic cell clusters. The color key from blue to red blue indicates low to high expression levels. PMC & IP, peritubular myoid cells & interstitial progenitors.

Supplementary Fig. 2 related to Fig. 2

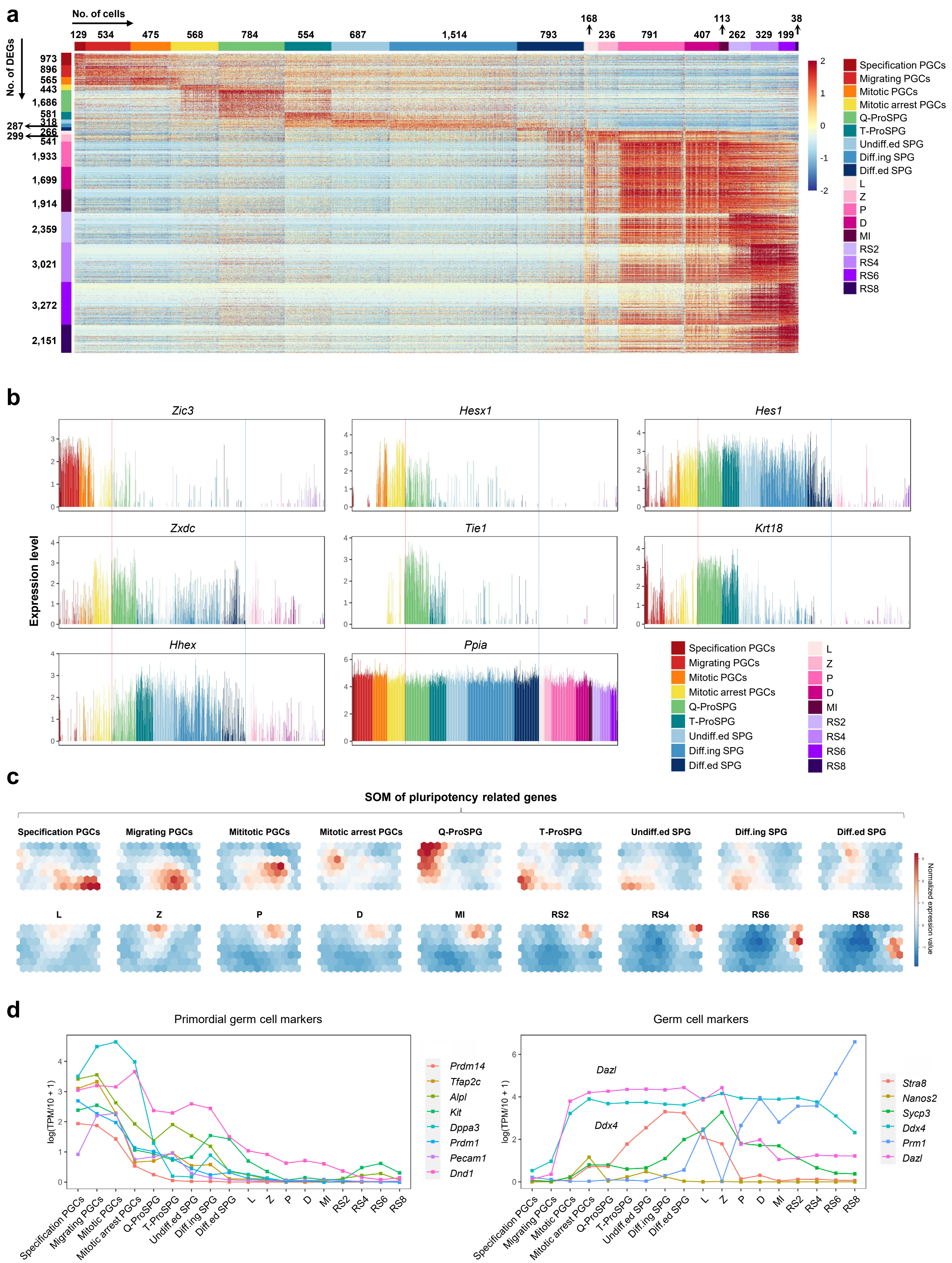

Supplementary Fig. 2 Global gene expression signature and gene expression dynamics of selected gene sets during mouse male germ cell development.

a, Heatmap of DEGs in 18 clusters of mouse male germ cells. Cell number and DEG number of each cluster are shown. The color key from blue to red indicates low to high gene expression levels. L, leptotene; Z, zygotene; P, pachytene; D, diplotene; MI, metaphase I; RS, round spermatids. **b,** Histograms showing the relative expression levels (log(TPM/10+1)) of genes representative for each cell cluster identified in this study. *Ppia* is a housekeeping gene. L, leptotene; Z, zygotene; P, pachytene; D, diplotene; MI, metaphase I; RS, round spermatids. **c,** Dynamic expression of pluripotency related genes represented by a self-organizing map algorithm; divergent expression patterns of pluripotency related genes are emerged in each cell cluster. A gradient of blue to red indicates low to high normalized expression value. L, leptotene; Z, zygotene; P, pachytene; D, diplotene; MI, metaphase I; RS, round spermatids. **d,** Line plots showing the relative expression levels (log(TPM/10+1)) of previously known PGC markers and germ cell markers in each cell cluster.

Supplementary Fig. 3 related to Fig. 2

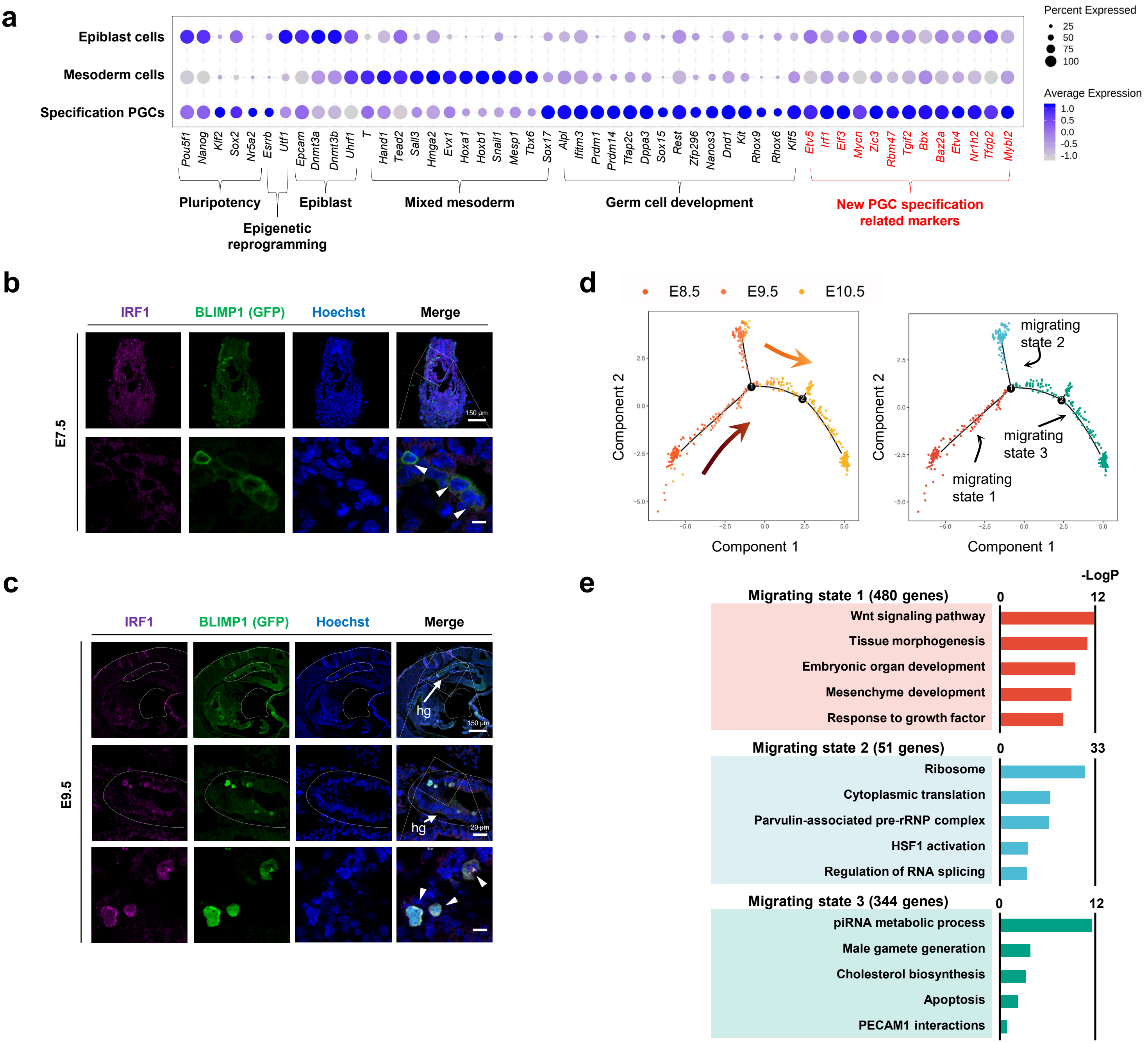

Supplementary Fig. 3 Transcriptional signatures of mouse specification PGCs and migrating PGCs.

a, Dotplot showing the expression patterns of gene sets in epiblast cells, mesoderm cells and specification PGCs sampled in this study. Dot size indicates the fraction of cells with detectable expression for a given marker gene and the color key indicates average gene expression levels in each cell type. **b,** Immunofluorescence of IRF1 co-stained with BLIMP1 (GFP) in E7.5 mouse embryos. Filled triangles indicate PGCs. Scale bar, 150 μm (labelled) and 10 μm (unlabelled). **c,** Immunofluorescence of IRF1 co-stained with BLIMP1 (GFP) in E9.5 mouse embryos. Arrows indicate hindgut, and filled triangles indicate PGCs. Scale bar, 150 μm (labelled), 20 μm (labelled), and 10 μm (unlabelled). **d,** Developmental pseudotime of migrating PGCs. (Left) Cells are colored by the sampled time-points are shown. (Right) Cells are colored by the three major migrating states. **e,** Enriched GO terms (performed by Metascape tool with well-adopted hypergeometric test and Benjamini-Hochberg *P*-value) of three migrating states.

Supplementary Fig. 4 related to Fig. 3

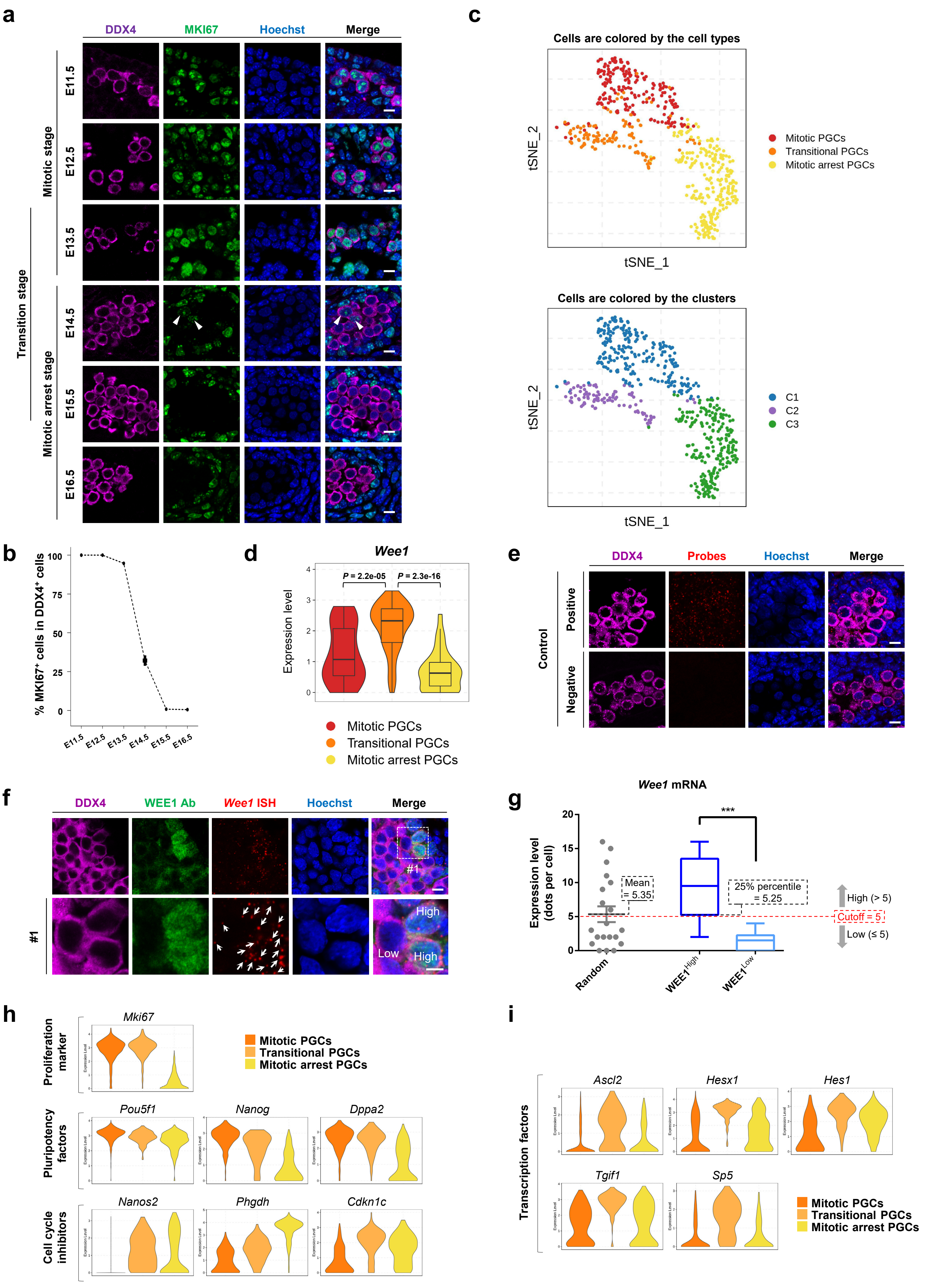

Supplementary Fig. 4 The identification and molecular characteristics of the transitional PGCs.

a, Immunofluorescence of the proliferation marker MKI67 co-stained with DDX4 in E11.5-E16.5 mouse male gonads. Scale bar, 10 μm. Arrowheads indicate the $Mki67^{+}$ PGCs at E14.5. **b,** Proportion of $DDX4^{+}$ cells that also stained for $MKI67^{+}$ at different developmental stages. Mean ± SEM, n = 4 per time-point. **c,** Dimension reduction and clustering analysis on the mitotic PGCs, transitional PGCs, and mitotic arrest PGCs at E13.5 to E15.5, when the mitotic-to mitotic arrest transition occurs. (Top) Dimension reduction and automatical clustering of the mitotic PGCs, transitional PGCs, and mitotic arrest PGCs. Cells are colored by the cell types. (Bottom) Dimension reduction and automatical clustering of the mitotic PGCs, transitional PGCs, and mitotic arrest PGCs. Cells are colored by the clusters. The mitotic PGCs, transitional PGCs, and mitotic arrest PGCs correspond to cluster C1, C2 and C3, respectively. **d,** Violin plot and box plot showing the expression level of *Wee1* in the mitotic PGCs, transitional PGCs and mitotic arrest PGCs, which can act as a marker of transitional PGCs. Centre line, median; box limits, interquartile range (IQR); whiskers, minima and maxima within 1.5 * IQR. Unpaired two-tailed wilcox test. (*P* values (< 0.0001) indicate the expression level of *Wee1* in the transitional PGCs is significantly higher than that of the mitotic PGCs and mitotic arrest PGCs). **e,** Positive and negative probes of RNAscope in E14.5 mouse male gonadal sections. These results indicate that the staining process and sections passed quality control measures. Scale bar, 10 μm. **f,** RNAscope of Wee1 (green) co-stained with immunofluorescence of DDX4 and WEE1 antibodies in E14.5 mouse male gonadal sections. Arrows indicate the dots of the probe signals, each representing one copy of the *Wee1* mRNA. Scale bar, 10 μm. Detailed images of the indicated germ cells are also shown. Scale bar, 5 μm. Images are obtained using ZEISS LSM880 confocal microscope under a C-Apochromat 63×/1.20 W korr M27 objective lens with 2× scan zoom. **g,** The *Wee1* mRNA staining signal dots in random, $WEE1^{High}$ (WEE1 protein high expression, detected by WEE1 antibody), and $WEE1^{Low}$ (WEE1 protein low expression, detected by WEE1 antibody) PGCs are quantitatively analyzed. In the left part, graphed results are presented as scatter dot plot and the mean dot counted per random PGC is 5.35. Mean ± SEM; In the right part, graphed results are presented as box and whiskers and the 25% percentile of the dots in $WEE1^{High}$ (WEE1 protein high expression, detected by WEE1

antibody) PGCs is 5.25. Centre line, median; box limits, interquartile range (IQR); whiskers, minima to maxima. P = 0.0001, unpaired two-tailed t test. We set 5 as cutoff to determine the "High" or "Low" expression level of *Wee1* mRNA (see Method). WEE1 protein and *Wee1* mRNA have the relatively consistent expression pattern. **h,** Violin plots showing the relative expression levels (log(TPM/10+1)) of representative genes related to pluripotency, cell cycle arrest and proliferation. **i,** Violin plots showing the relative expression levels (log(TPM/10+1)) of representative transcription factors highly expressed in transitional PGCs.

Supplementary Fig. 5 related to Fig. 3

a

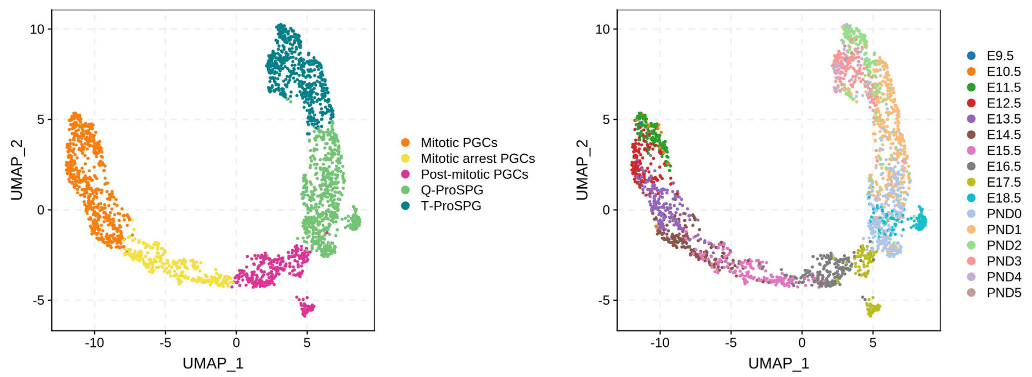

b

Mitotic PGCs	-LogP
Metabolism of RNA	37
Cell Cycle	34
Ribonucleoprotein complex biogenesis	25
Mitotic cell cycle process	24
Translation	23

Transitonal PGCs	-LogP
Chromosome segregation	26
Meiotic nuclear division	14
Regulation of APC/C activators between G1/S and early anaphase	11
Cell cycle	11
Spindle organization	11

Mitotic arrest PGCs	-LogP
ncRNA metabolic process	7.0
Chromatin organization	5.2
Macromolecule methylation	4.9
DNA methylation involved in gamete generation	4.5
Organelle fission	4.4

Post-arrest PGCs	-LogP
DNA methylation involved in gamete generation	10
Cilium assembly	6.9
Cellular process involved in reproduction in multicellular organism	6.4
Actin filament-based process	4.6
G1 to G0 transition	3.7

Q-ProSPG	-LogP
Behavior	9.3
Regulation of neuron projection development	9.1
Regulation of secretion by cell	8.3
Supramolecular fiber organization	7.0
Developmental Biology	6.7

T-ProSPG	-LogP
Actin cytoskeleton organization	8.5
Axon guidance	7.8
Rho protein signal transduction	7.3
Synapsis	6.8
Osteoclast differentiation	6.4

c

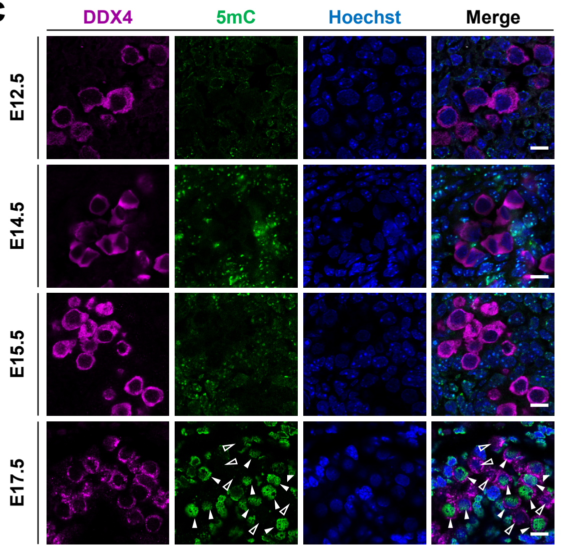

d

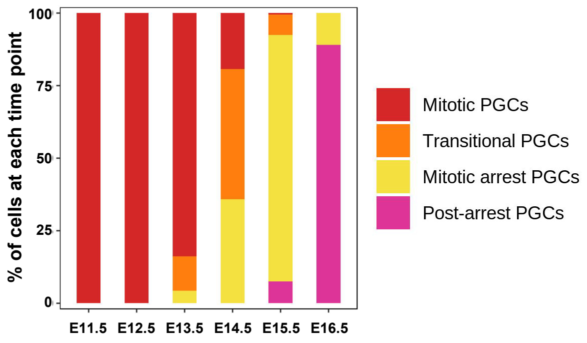

e

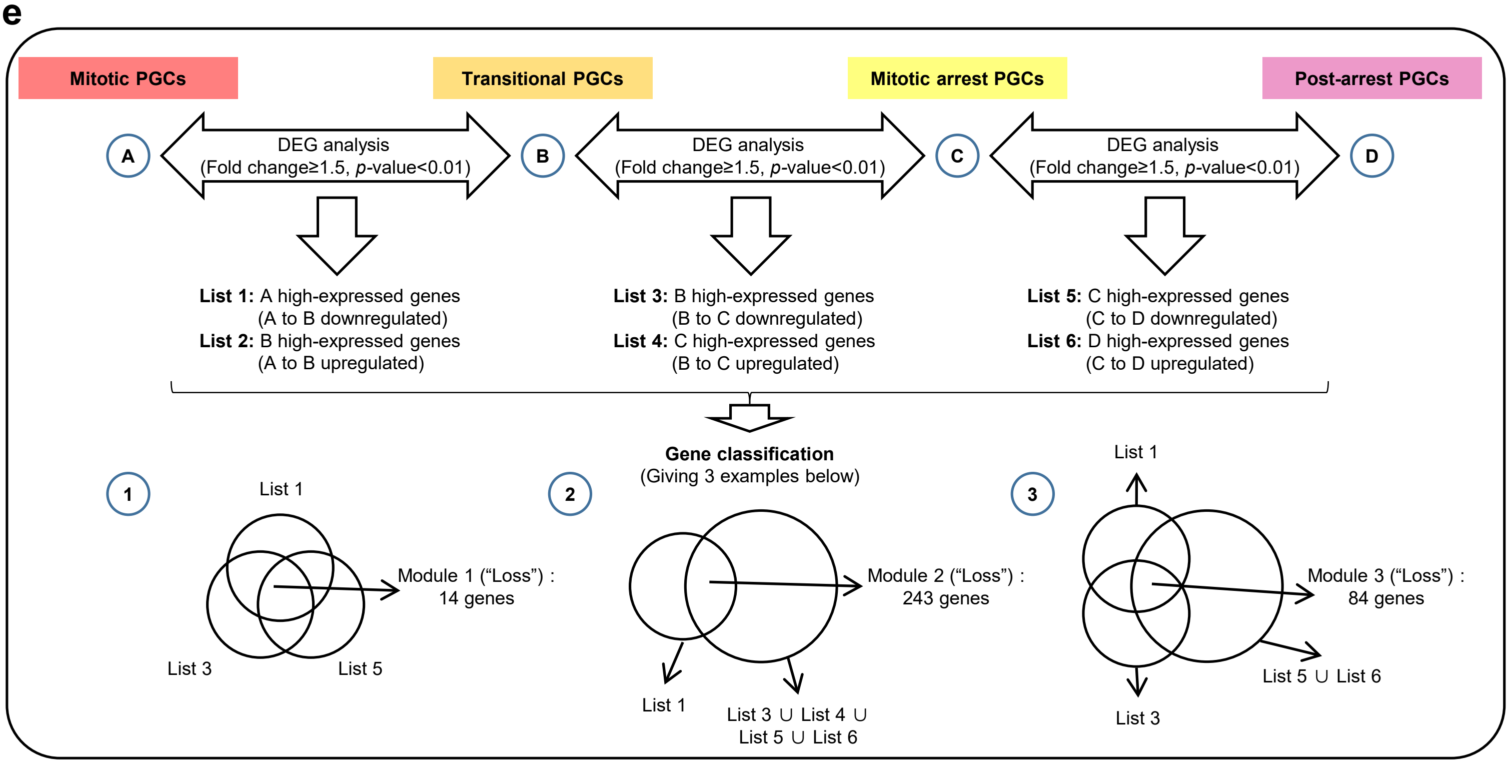

Supplementary Fig. 5 Gene expression signature and GO analyses of intermediate cell states during mouse male germ cell development.

a, (Left) UMAP plot of the mitotic PGCs, mitotic arrest PGCs, post-arrest PGCs, Q-ProSPG, and T-ProSPG. Cells are colored by indicated cell types. (Right) UMAP plot of the mitotic PGCs, mitotic arrest PGCs, post-arrest PGCs, Q-ProSPG, and T-ProSPG. Cells are colored by indicated developmental time-points. **b,** Enriched GO terms and *P* values (-LogP) of the mitotic PGCs, transitional PGCs, mitotic arrest PGCs, post-arrest PGCs, Q-ProSPG, and T-ProSPG. **c,** Immunofluorescence of 5mC (5-Methylcytosine) co-stained with DDX4 in E12.5, E14.5, E15.5, and E17.5 mouse male gonads. Hollow and solid white arrowheads indicate $5mC^{Negative}$ and $5mC^{Positive}$ subtypes in $DDX4^{+}$ cells, respectively. Scale bar, 10 μm. **d,** Relative distributions of mitotic-, transitional-, mitotic arrest-, and post-arrest PGCs from E11.5 to E16.5. **e,** Schematic diagram of clustering analysis of dynamic gene expression during mitotic to mitotic arrest transition.

Supplementary Fig. 6 related to Fig. 4

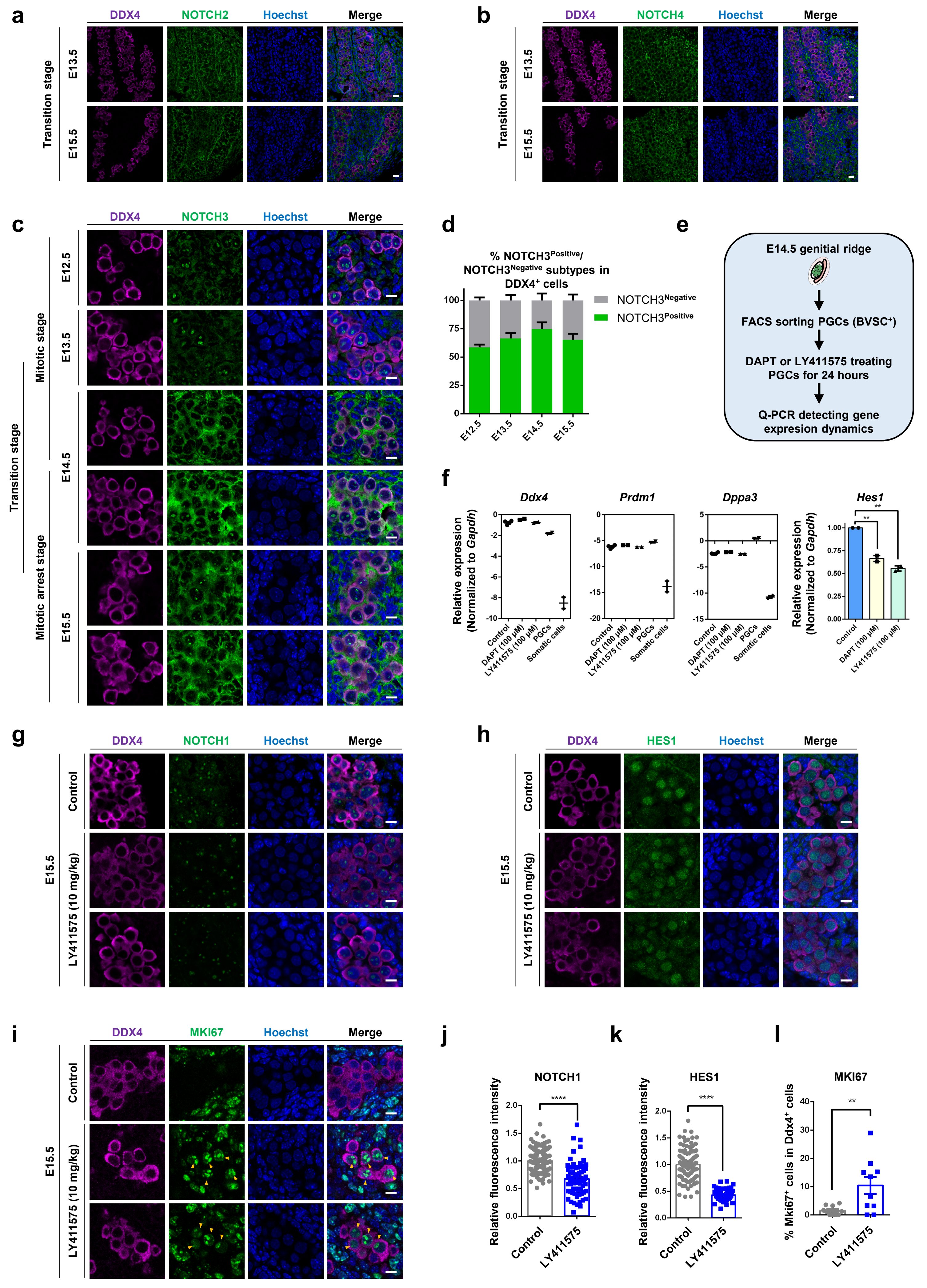

Supplementary Fig. 6 Detection of Notch signaling pathway components and Notch signaling pathway blocking *in vitro* or *in vivo*.

a, Immunofluorescence of NOTCH2 co-stained with DDX4 in E13.5 and E15.5 mouse male gonads. Scale bar, 10 μm. **b,** Immunofluorescence of NOTCH4 co-stained with DDX4 in E13.5 and E15.5 mouse male gonads. Scale bar, 10 μm. **c,** Immunofluorescence of NOTCH3 co-stained with DDX4 in E12.5-E15.5 mouse male gonads. Scale bar, 10 μm. **d,** Proportion of $NOTCH3^{Negative}$ and $NOTCH3^{Positive}$ subtypes in $DDX4^{+}$ cells in E13.5 and E15.5 mouse male gonads. Mean ± SEM, n = at least 3 biologically independent samples for each time-point. **e,** Schematic showing the workflow of treatment by γ-Secretase inhibitor−DAPT or LY411575 for Notch signaling pathway blocking *in vitro*. **e,** Schematic showing the workflow of treatment by γ-Secretase inhibitor−DAPT or LY411575 for Notch signaling pathway blocking *in vitro*. The $BVSC^{+}$ cells (PGCs) were cultured in a well of 5,000 cells in a low-cell-binding U-bottom 96-well plate in PGC medium (see Method). **f,** Q-PCR analysis of the expression of PGCs marker genes (*Ddx4*, *Prdm1*, *Dppa3*) and Notch signaling pathway target gene *Hes1* in *in vitro* cultured PGCs after 24 h treated by DAPT or LY411575. To verify the germ cell identity, the sorted PGCs and somatic cells without treatment are used as the controls. Relative expression levels are shown with normalization to *Gapdh*. Error bars indicate mean ± SEM from at least 2 independent biological replicates. ** $P < 0.01$, Control vs. DAPT ($P = 0.0091$), Control vs. LY411575 ($P = 0.0039$), unpaired two-tailed t test. **g,** Immunofluorescence of NOTCH1 co-stained with DDX4 in LY411575-treatment and control mouse male gonads at E15.5. Scale bar, 10 μm. **h,** Immunofluorescence of HES1 co-stained with DDX4 in LY411575-treatment and control mouse male gonads at E15.5. Scale bar, 10 μm. **i,** Immunofluorescence of MKI67 co-stained with DDX4 in LY411575-treatment and control mouse male gonads at E15.5. Solid yellow arrowheads indicate $MKI67^{Positive}$ subtypes in $DDX4^{+}$ cells. Scale bar, 10 μm. **j,** The quantification of relative fluorescence intensity of NOTCH1 in LY411575-treatment and control mouse male gonads at E15.5. Mean ± SEM, n = 3 per group, **** $P < 0.0001$, P = 1.4E-17, unpaired two-tailed t test. **k,** The quantification of relative fluorescence intensity of HES1 in LY411575-treatment and control mouse male gonads at E15.5. Mean ± SEM, n = 3 per group, **** $P < 0.0001$, P = 3.7E-35, unpaired two-tailed t test. **l,** Proportion of $MKI67^{+}$

cells in $DDX4^+$ cells in LY411575-treatment and control mouse male gonads at E15.5. Mean ± SEM, n = 3 per group, ** $P < 0.01$, $P = 0.0085$, unpaired two-tailed t test.

Supplementary Fig. 7 related to Fig. 5

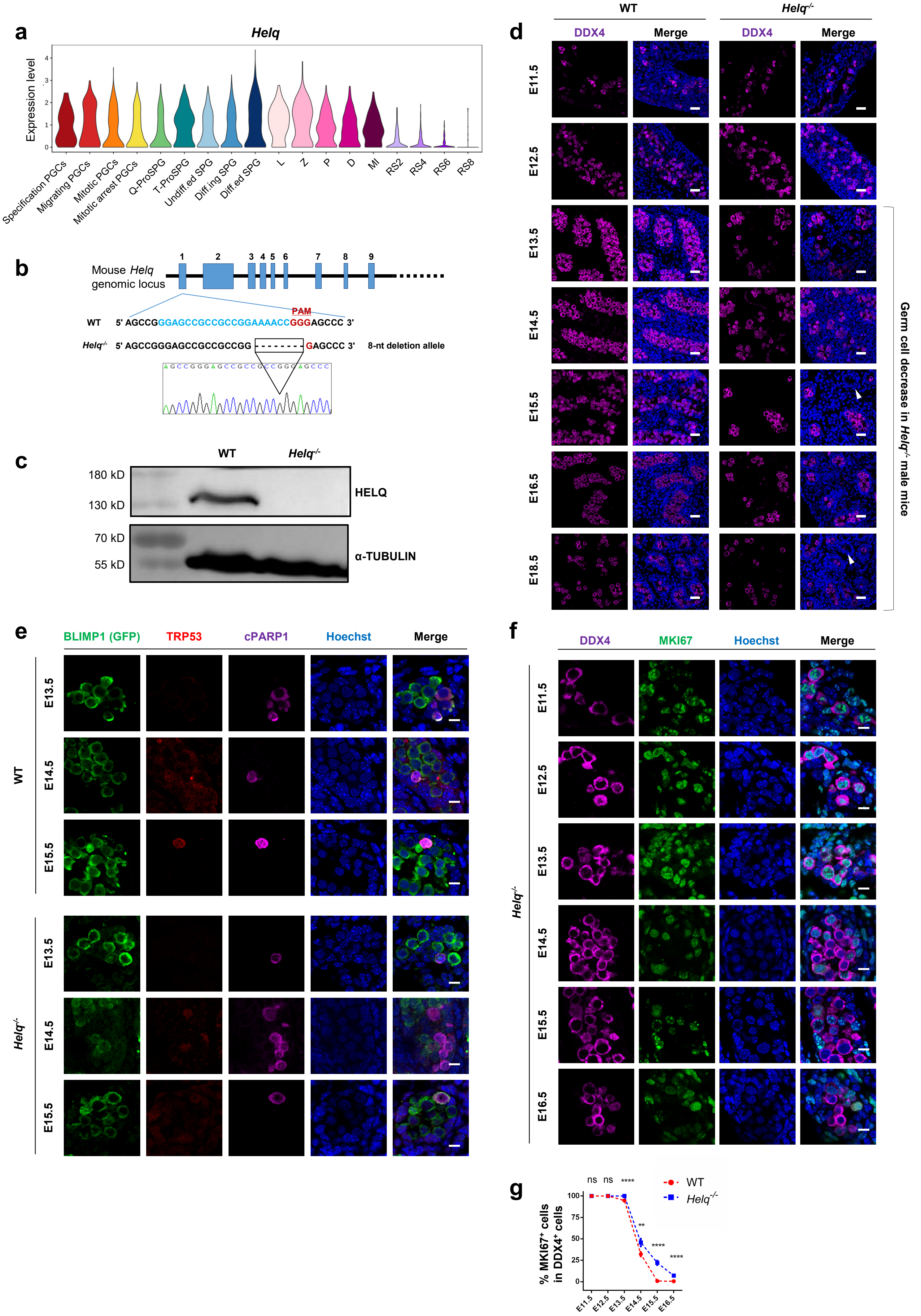

Supplementary Fig. 7 Immunostaining analysis of *Helq*$^{-/-}$ mice.

a, Violin plot showing the relative expression levels (log(TPM/10+1)) of *Helq* in all mouse male germ cell clusters. **b,** (Top) *Helq* genomic locus showing the design of sgRNA targeted to exon 1. (Bottom) Sanger sequencing showing the 8-nt deletion in the two alleles of *Helq* in *Helq*$^{-/-}$ mouse. **c,** Western blot analysis of the expression HELQ protein in *Helq*$^{-/-}$ mice testis, α-TUBULIN as the inner control. Source data are provided as a Source Data file. **d,** Immunofluorescence of DDX4 from E11.5 to E16.5, and E18.5 in the WT- and *Helq*$^{-/-}$ mouse male gonads, merged with Hoechst (blue). Scale bar, 25 μm. Arrowheads indicate the empty lumen in the gonads. **e,** Immunofluorescence of TRP53 (red) and cPARP1 (pink) co-stained with BLIMP1 (GFP, green) in E13.5, E14.5 and E15.5 from the WT- and *Helq*$^{-/-}$ mouse male gonads. Scale bar, 10 μm. **f,** Immunofluorescence of MKI67 co-stained with DDX4 in E11.5-E16.5 *Helq*$^{-/-}$ mouse male gonads. Scale bar, 10 μm. **g,** Proportions of MKI67^{+} cells co-staining with DDX4^{+} from E11.5 to E16.5, in the WT- and *Helq*$^{-/-}$ mouse male gonads. Mean ± SEM, n = 4 per time-point, ns, not significant, ** $P < 0.01$, **** $P < 0.0001$, all the values included in statistics at E11.5 and E12.5 are 100% for WT and *Helq*$^{-/-}$ genotypes, E13.5 (P = 3.5E-05), E14.5 (0.0023), E15.5 (5.7E-06), and E16.5 (1.3E-08), unpaired two-tailed t test.

Supplementary Fig. 8 related to Fig. 5

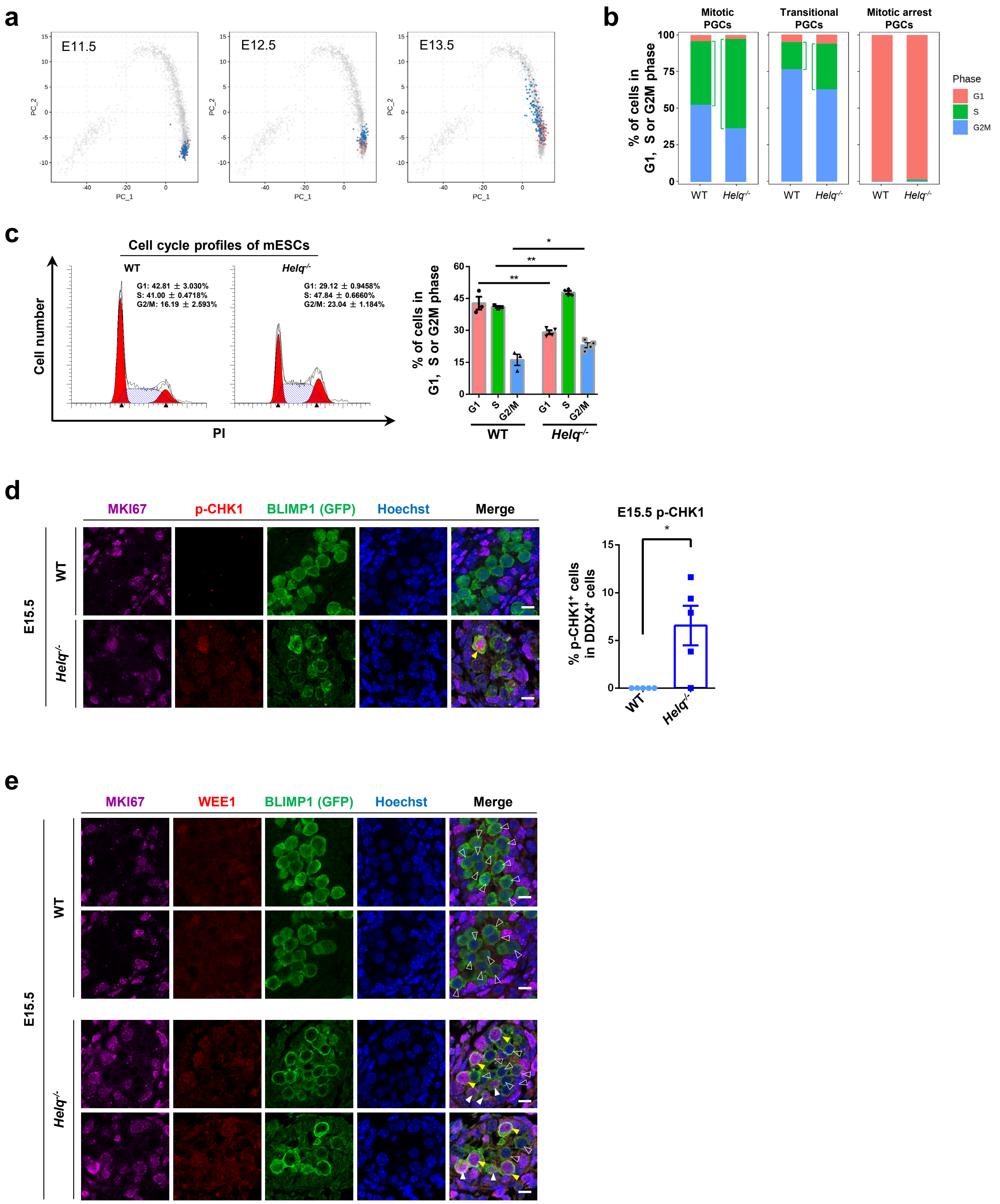

Supplementary Fig. 8 Transcriptome analysis of *Helq*$^{-/-}$ mice.

a, PCA (Principal component analysis) plots showing the developmental trajectory of 985 WT- and 724 *Helq*$^{-/-}$ mouse male germ cells sampled between E11.5 and E13.5. The same PCA is also plotted, highlighting only cells from each stage (cell numbers: n = 151, WT; n = 72, *Helq*$^{-/-}$ for E11.5; n = 137, WT; n = 70, *Helq*$^{-/-}$ for E12.5; n = 185, WT; n = 132, *Helq*$^{-/-}$ for E13.5; n = 187, WT; n = 191, *Helq*$^{-/-}$ for E14.5; n = 185, WT; n = 187, *Helq*$^{-/-}$ for E15.5; n = 140, WT; n = 72, *Helq*$^{-/-}$ for E18.5, also see Fig. 5c). **b,** Proportion germ cells of three cell cycle phases in the WT- versus *Helq*$^{-/-}$ mouse male gonads. **c,** Cell cycle profiles of the WT- and *Helq*$^{-/-}$ mouse embryonic stem cells determined by flow cytometry (left). Cell cycle profiles were presented as the mean of five independent experiments (right). Quantitative data are shown as the mean ± SEM, n = 3 and 4 for WT and *Helq*$^{-/-}$ group, respectively, * $P < 0.05$, ** $P < 0.01$, $P = 0.0043$, 0.0010, and 0.0453 for comparison of cell cycle ratio at G1, S, and G2M phase, respectively, unpaired two-tailed t test. **d,** (Left) Immunofluorescence of p-CHK1 co-stained with MKI67 and BLIMP1 (GFP) and (Right) the relative proportions of of p-CHK1^{+} germ cells in the WT- and *Helq*$^{-/-}$ mouse male gonads at E15.5. Scale bar, 10 μm. Arrowheads indicate p-CHK1^{+} subtypes in MKI67^{+} germ cells. Mean ± SEM, n = 4 per group, * $P < 0.05$, $P = 0.0134$, unpaired two-tailed t test. **e,** Immunofluorescence of WEE1 co-stained with MKI67 and BLIMP1 (GFP) in the WT- and *Helq*$^{-/-}$ mouse male gonads at E15.5. Scale bar, 10 μm.

Supplementary Fig. 9 related to Fig. 5

a

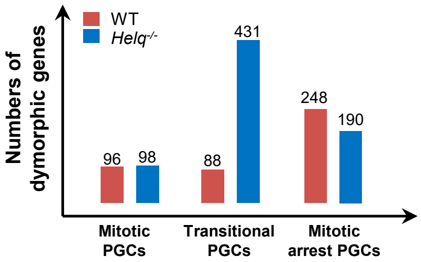

b

Upregulated in *Helq*$^{-/-}$ transitional PGCs	-LogP
Hippo signaling pathway	8.8
Phosphatidylinositol signaling system	8.2
Pathways in cancer	7.6
Blood vessel morphogenesis	7.3
Dephosphorylation	7.1
DNA integrity checkpoint	4.0
DNA damage checkpoint	2.9

Downregulated in *Helq*$^{-/-}$ mitotic arrest PGCs	-LogP
Cilium organization	4.6
Antigen processing and presentation	3.9
Positive regulation of autophagy	3.4
Toll-like receptor 3 signaling pathway	3.1
Flagellated sperm motility	3.0
Rho protein signal transduction	2.8
Regulation of endopeptidase activity	2.8

c

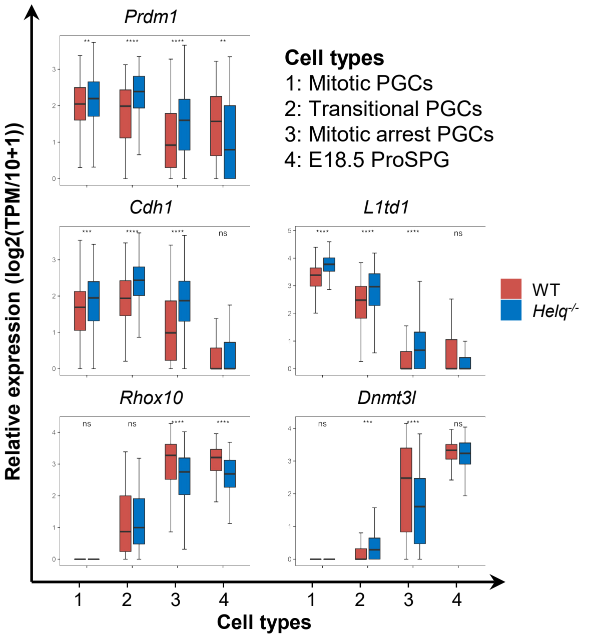

d

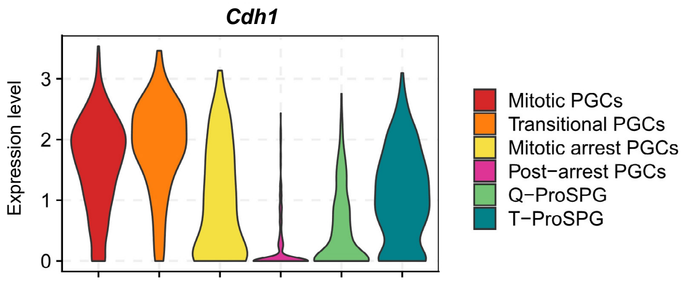

e

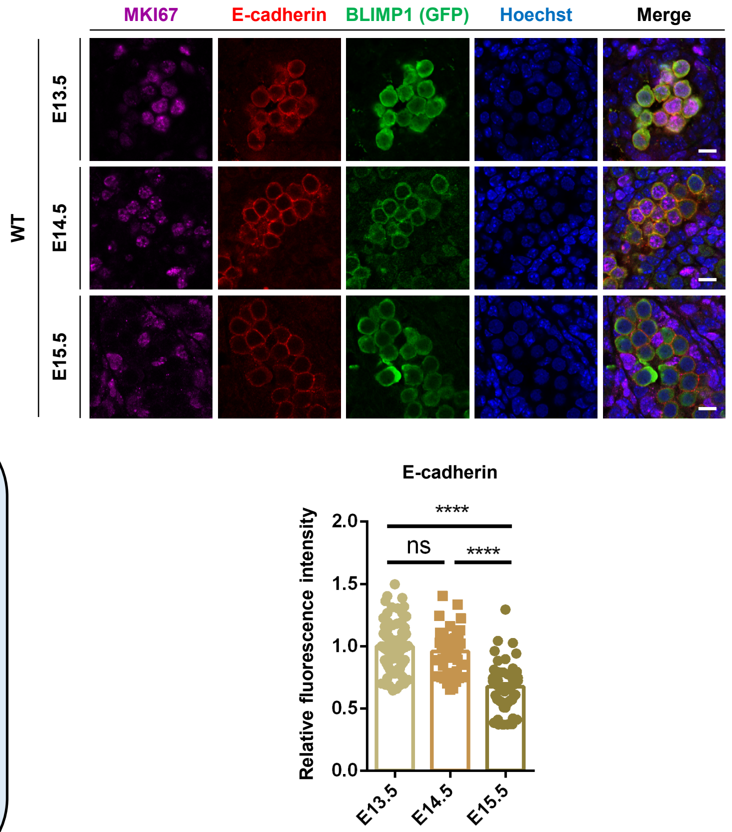

f

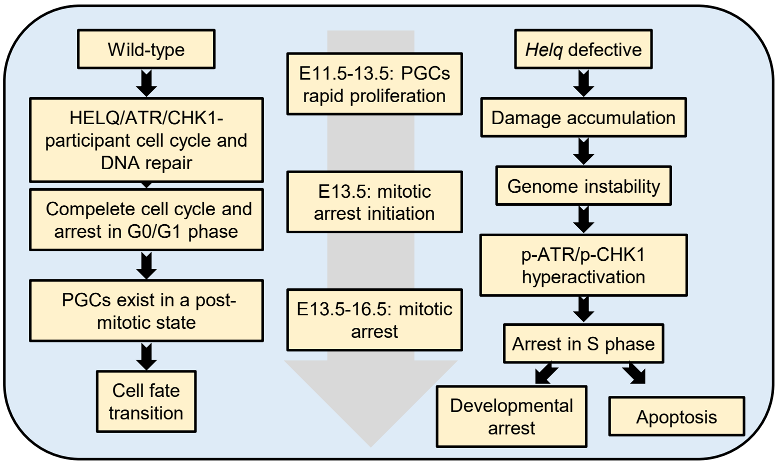

Supplementary Fig. 9 Downstream analysis based on scRNA-seq data and immunofluorescence verification of candidate downstream targets.

a, Numbers of the genes up/downregulated in the WT- versus *Helq*$^{-/-}$ PGCs. **b,** Representative GO terms (performed by Metascape tool with well-adopted hypergeometric test and Benjamini-Hochberg *P*-value) of the genes upregulated and downregulated in *Helq*$^{-/-}$ transitional- and mitotic arrest PGCs, respectively. **c,** Boxplot showing expression level of representative misregulated marker genes. Centre line, median; box limits, IQR; whiskers, minima and maxima within 1.5 * IQR, ns, not significant, n = at least 72 cells per cell population examined over two biologically independent experiments, ns, not significant, * $P < 0.05$, ** $P < 0.01$, *** $P < 0.001$ and **** $P < 0.0001$, unpaired two-tailed wilcox test. **d,** Violin plots showing expression levels of *Cdh1* at different stages in the WT mouse male germ cells. **e,** (Top) Immunofluorescence of E-cadherin co-stained with MKI67 and BLIMP1 (GFP) in the WT mouse male gonads at E13.5, E14.5, and E15.5. Scale bar, 10 μm. (Bottom) The quantification of relative fluorescence intensity of E-cadherin in the WT mouse male germ cells at E13.5, E14.5, and E15.5. Mean ± SEM, ns, not significant, **** $P < 0.0001$, E13.5 vs. E14.5 ($P = 0.2843$), E13.5 vs. E15.5 ($P = 2.0E-15$), E14.5 vs. E15.5 ($P = 1.2E-12$), unpaired two-tailed t test. **f,** Illustration of the mouse male germ cells development in HELQ defective condition.

Supplementary Fig. 10 related to Fig. 6

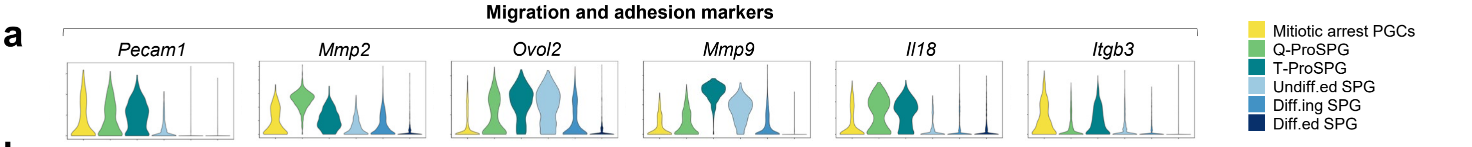

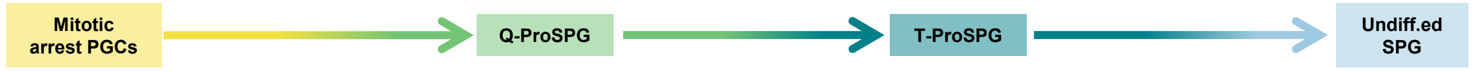

Downregulated genes: 281	-LogP
Cell Cycle Checkpoints	21
Mitotic cell cycle process	15
APC/C-mediated degradation of cell cycle proteins	11
Microtubule cytoskeleton organization involved in mitosis	9.0
G2/M Transition	8.5

Upregulated genes: 1063	-LogP
Supramolecular fiber organization	8.9
Formation of the cornified envelope	7.7
Behavior	7.2
Regulation of system process	7.0
Regulation of neuron projection development	6.0

Downregulated genes: 1099	-LogP
Cilium movement	9.7
Behavior	6.6
Reflex	5.0
Skeletal system development	4.9
Cellular response to metal ion	4.5

Upregulated genes: 528	-LogP
Cell Cycle	39
DNA replication	18
M Phase	18
Mitotic cell cycle process	17
Mitotic G1 phase and G1/S transition	14

Downregulated genes: 293	-LogP
Regulation of system process	8.4
Regulation of transmembrane transport	6.4
Actin filament-based process	6.2
Striated muscle adaptation	5.6
Response to metal ion	5.0

Upregulated genes: 42	-LogP
Cell division	4.7
Stem cell division	4.2
Positive regulation of canonical Wnt signaling pathway	3.3
Connective tissue development	3.1
Metabolism of carbohydrates	2.2

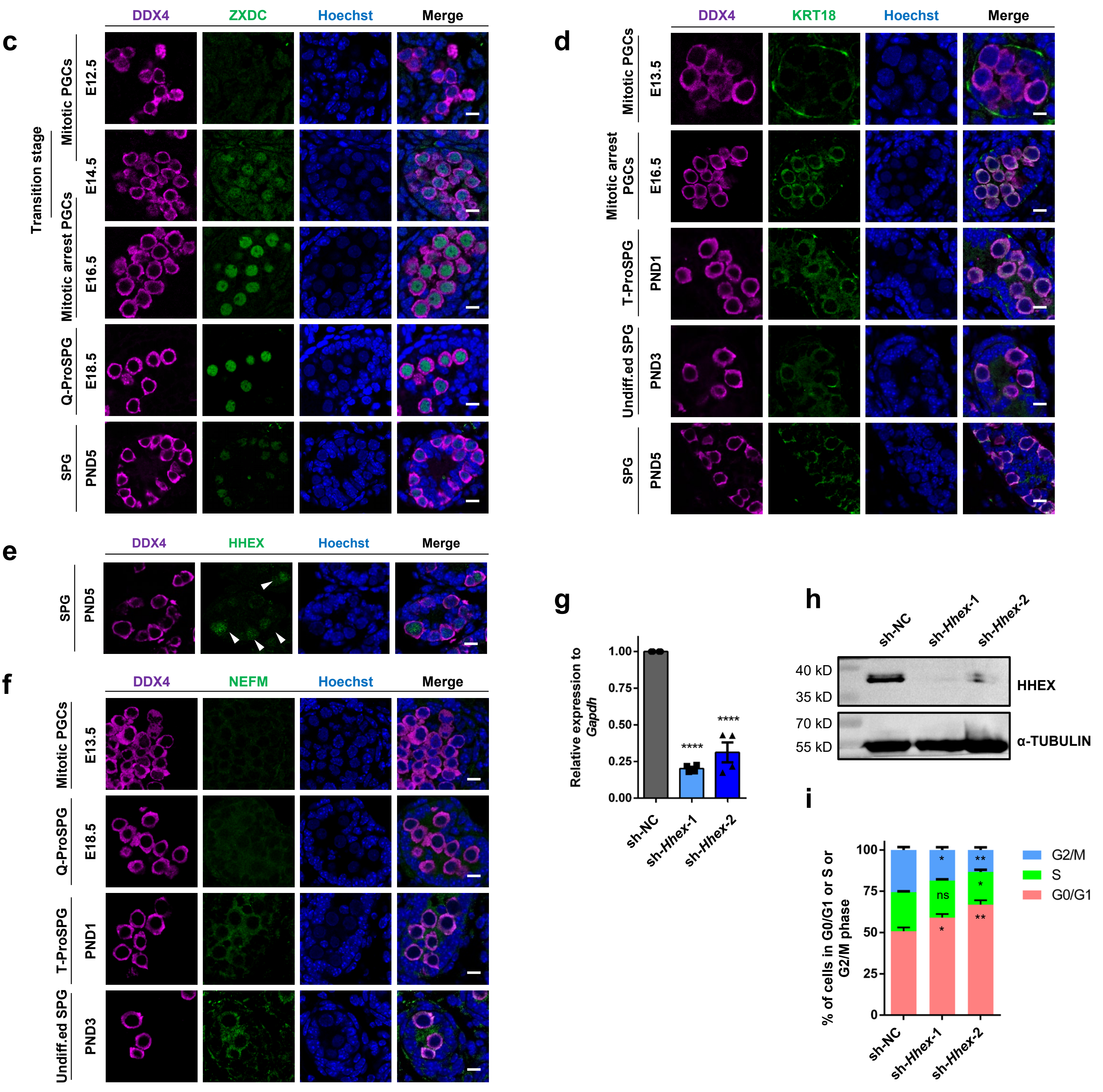

Supplementary Fig. 10 GO analysis and gene expression signature of prospermatogonia/ spermatogonia and immunostaining analysis of selected markers.

a, Violin plots showing the relative expression levels (log(TPM/10+1)) of representative genes associated with cell migration and adhesion. **b,** Enriched GO terms of developmental transitions, based on mitotic arrest PGCs, Q-ProSPG, T-ProSPG, and undiff.ed SPG. **c,** Immunofluorescence of ZXDC co-stained with DDX4 in E12.5, E14.5, E16.5, E18.5, and PND5 mouse testis. Scale bar, 10 µm. **d,** Immunofluorescence of KRT18 co-stained with DDX4 in E13.5, E16.5, PND1, PND3, and PND5 mouse testis. Scale bar, 10 µm. **e,** Immunofluorescence of HHEX co-stained with DDX4 in PND5 mouse testis. Scale bar, 10 µm. **f,** Immunofluorescence of NEFM co-stained with DDX4 in E13.5, E18.5, PND1, and PND3 mouse testis. Scale bar, 10 µm. **g,** Quantitative analysis of *Hhex* mRNA 24 h after transfection with non-targeting shRNA (sh-NC) and shRNAs against *Hhex* in mSSCs. Data are presented as the mean ± SEM, n = 3, **** $P < 0.0001$, sh-NC vs. sh-*Hhex*-1 (P = 6.5E-10), sh-NC vs. sh-*Hhex*-2 (P = 5.3E-05), unpaired two-tailed t test. **h,** Verification of knockdown efficiency in mSSCs by western blot analysis, 24 h after transfection with non-targeting shRNA or shRNAs against *Hhex* in mSSCs, α-TUBULIN was used as the inner control. Source data are provided as a Source Data file. **i,** Cell cycle analysis of *Hhex*-knockdown mSSCs. Quantitative data are shown as the mean ± SEM, n = at least 4, ns, not significant, * $P < 0.05$, ** $P < 0.01$, G0/G1 phase: sh-NC vs. sh-*Hhex*-1 (P = 0.0328), sh-NC vs. sh-*Hhex*-2 (P = 0.0021); S phase: sh-NC vs. sh-*Hhex*-1 (P = 0.2486), sh-NC vs. sh-*Hhex*-2 (P = 0.0175); G2/M phase: sh-NC vs. sh-*Hhex*-1 (P = 0.0205), sh-NC vs. sh-*Hhex*-2 (P = 0.0015), unpaired two-tailed t test.

Supplementary Fig. 11 related to Fig. 6

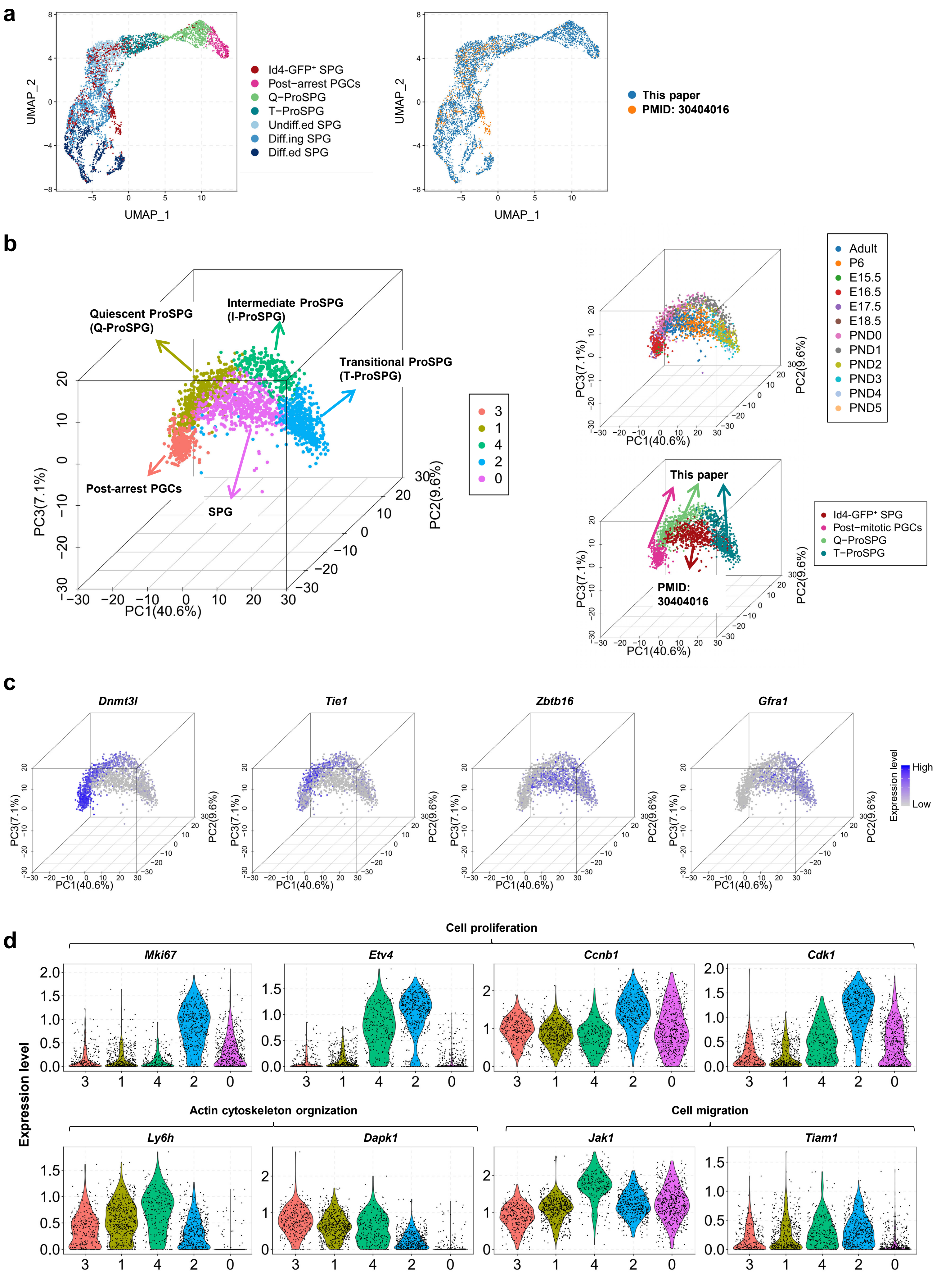

Supplementary Fig. 11 Integration analysis of mouse male germ cell development from this study and a previous study (PMID 30404016).

a, UMAP plots showing integration results of post-arrest PGCs, Q-ProSPG, T-ProSPG, undiff.ed SPG, diff.ing SPG, and diff.ed SPG from this study and 578 Id4-GFP$^+$ spermatogonia from Hermann *et al* (PMID 30404016). Cells are colored based on cell types (left) and data sources (right) are shown. **b,** 3D PCA plots showing integration results of post-arrest PGCs, Q-ProSPG, T-ProSPG from this study and 578 Id4-GFP$^+$ spermatogonia cells from Hermann *et al* (PMID 30404016). Cells are colored based on cell types (left), time points (upper right) and data sources (lower right) are shown. **c,** 3D PCA plots of *Dnmt3l*, *Tie1*, *Zbtb16*, and *Gfra1*. The color key from gray to blue indicates low to high expression levels. **d,** Violin plots showing expression levels of cell proliferation-related markers, actin cytoskeleton organization genes and cell migration-related markers in each cluster of integration result between this study and Hermann *et al* (PMID 30404016).

Supplementary Fig. 12 related to Fig. 6

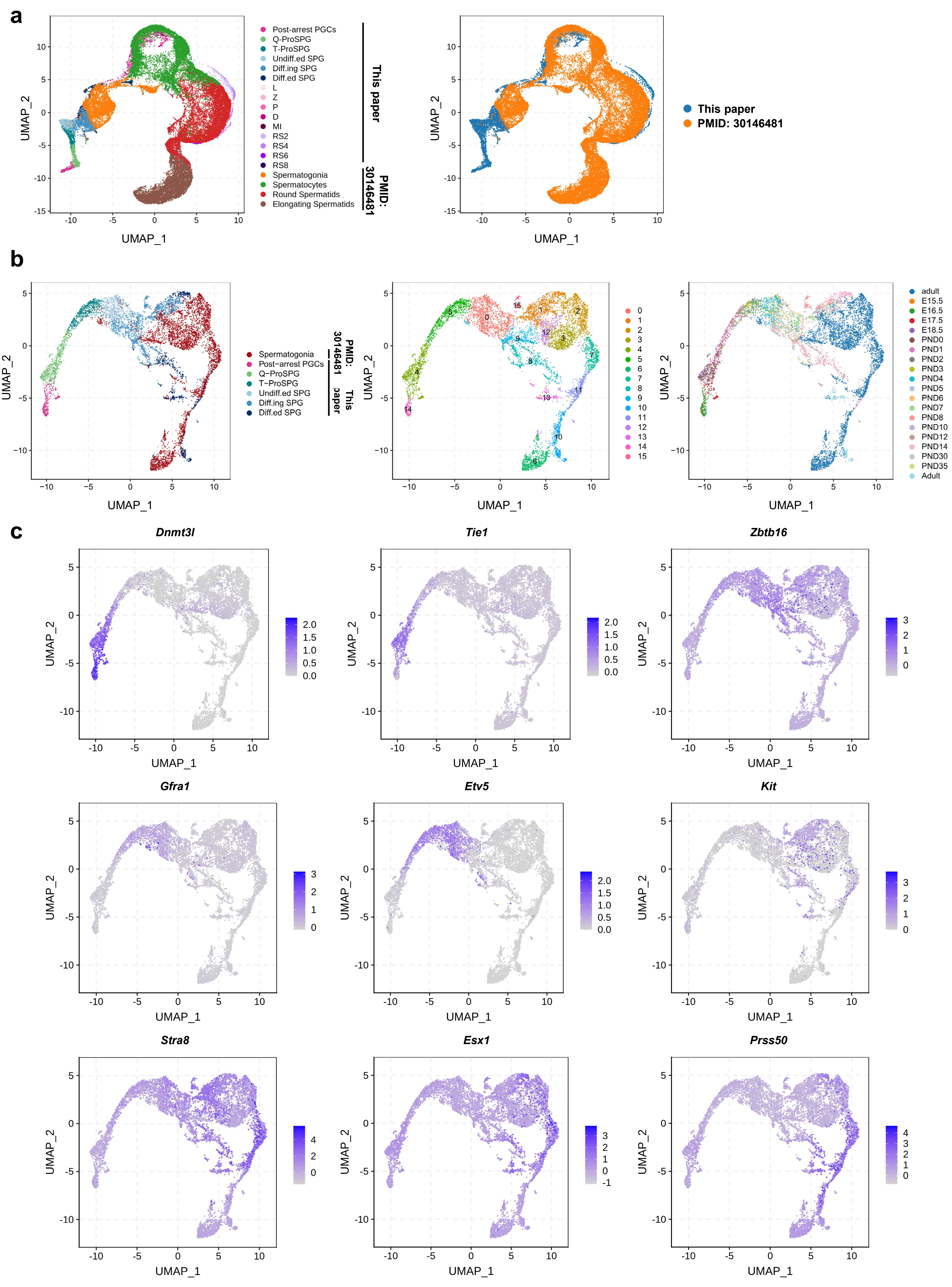

Supplementary Fig. 12 Integration analysis of mouse male germ cell development from this study and a previous study (PMID 30146481).

a, UMAP plots showing integration results of germ cells from this study and Green *et a*l. (PMID 30146481). Cells are colored based on cell types (left) and references (right). **b,** UMAP plots showing integration results of germ cells from this study and Green *et al* (PMID 30146481). Cells are colored based on cell types (left), clusters (middle) and time points (right). **c,** UMAP plots of germ cell markers. The color key from gray to blue indicates low to high expression levels.

Supplementary Fig. 13

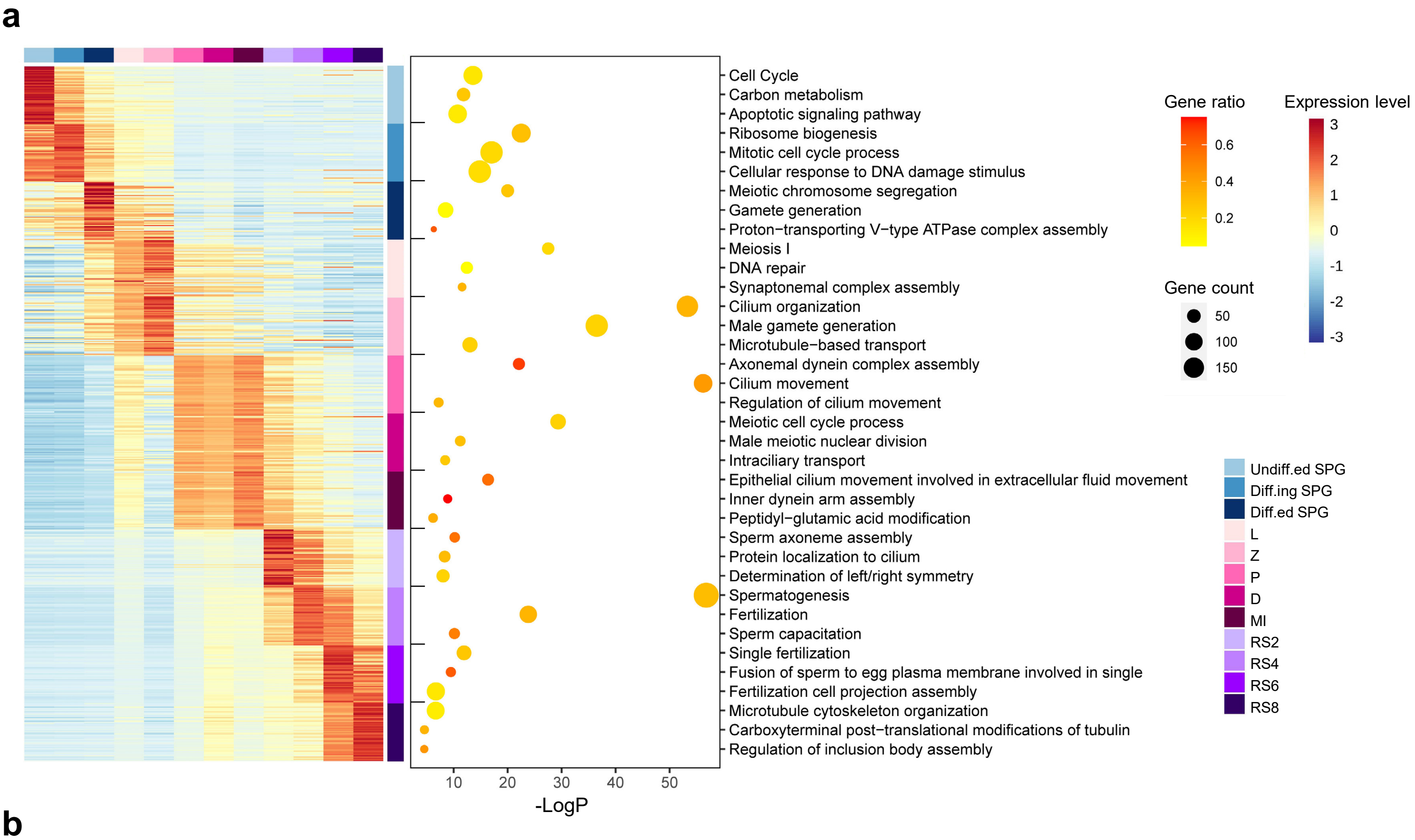

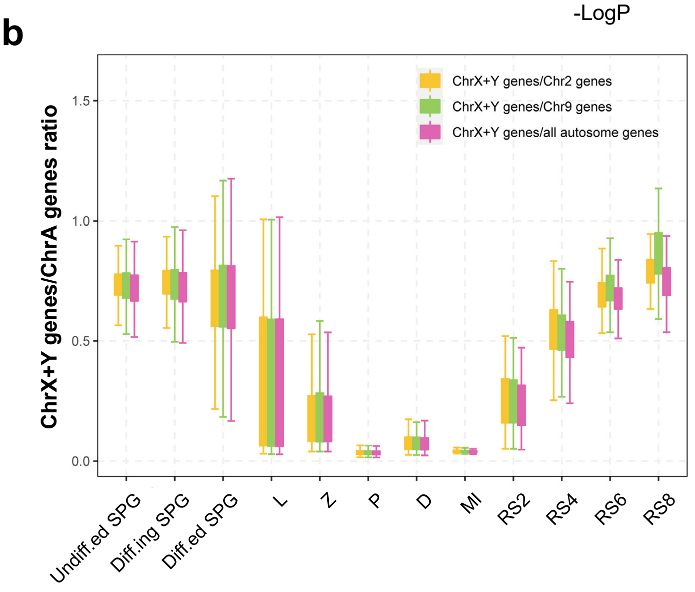

Supplementary Fig. 13 Transcriptome dynamics from SPG to SPC and further to RS.

a, (Left) Heatmap of the top 50 DEGs in 12 cell types of mouse male germ cell development. The color key from blue to red indicates low to high gene expression levels. L, leptotene; Z, zygotene; P, pachytene; D, diplotene; MI, metaphase I; RS, round spermatids. (Right) Bubble plot shows top enriched GO terms of 12 cell type of mouse male germ cell development. The size and color of bubbles represent the number of genes and gene ratio in corresponding GO terms, respectively. **b,** Boxplot showing the ratio of average expression level of selected genes between two classes of chromosomes. Yellow represents the ratio of gene expression level between sex chromosomes and chromosome 2. Prasinous represents the ratio of gene expression level between sex chromosomes and chromosome 9. Rose represents the ratio of gene expression level between sex chromosomes and autosome. Centre line, median; box limits, IQR; whiskers, minima and maxima within 1.5 * IQR.

Supplementary Fig. 14 related to Fig. 7

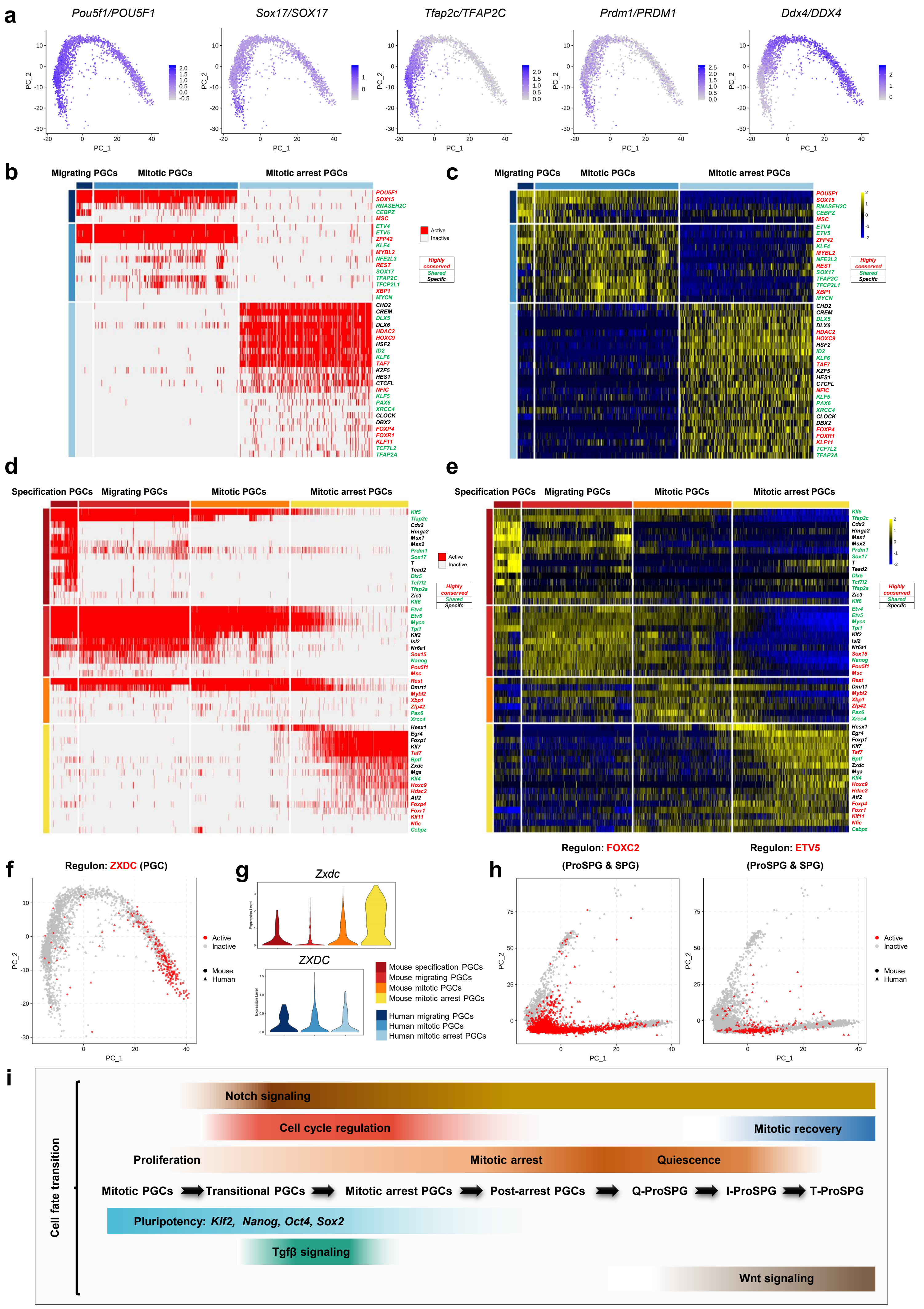

Supplementary Fig. 14 Conserved differentially expressed genes dissection and SCENIC analysis in both PGCs and spermatogonia between human and mouse.

a, Gene expression patterns of maker genes on PCA plots related to Fig. 7a,b. The color key from gray to blue indicates low to high expression levels. **b,** SCENIC results for the human PGCs. Heatmap showing the active and expressed regulators in each cell class. The states of the transcription factors in each cell class are indicated in red (active) and grey (inactive). The highly conserved DEGs, human-mouse shared DEGs and species-specific DEGs are labelled by red, green and black, respectively. **c,** Heatmap showing the expression of the active transcription factors in each human cell cluster. Expression levels are indicated with colors. The highly conserved DEGs, human-mouse shared DEGs and species-specific DEGs are labelled by red, green and black, respectively. **d,** SCENIC results for the mouse PGCs. Heatmap showing the active and expressed regulators in each cell class. The states of the transcription factors in each cell class are indicated in red (active) and grey (inactive). The highly conserved DEGs, human-mouse shared DEGs and species-specific DEGs are labelled by red, green and black, respectively. **e,** Heatmap showing the expression of the active transcription factors in each mouse cell cluster. Expression levels are indicated with colors. The highly conserved DEGs, human-mouse shared DEGs and species-specific DEGs are labelled by red, green and black, respectively. **f,** The regulon activity PCA map of Zxdc/ZXDC in mouse and human PGCs; red dots represent active state of the regulators in each cell, and the light grey dots represent inactive state of the regulators in each cell. **g,** Violin plots showing the relative expression levels (log(TPM/10+1)) of *Zxdc/ZXDC* in mouse and human PGCs. **h,** The regulon activity PCA map of Foxc2/FOXC2 and Etv5/ETV5 in mouse ProSPG and SPG and human SPG; red dots represent active state of the regulators in each cell, and the light grey dots represent inactive state of the regulators in each cell. **i,** Sketch of the expression patterns of different set of genes and signaling pathways during mouse germ cell development.

Supplementary Fig. 15

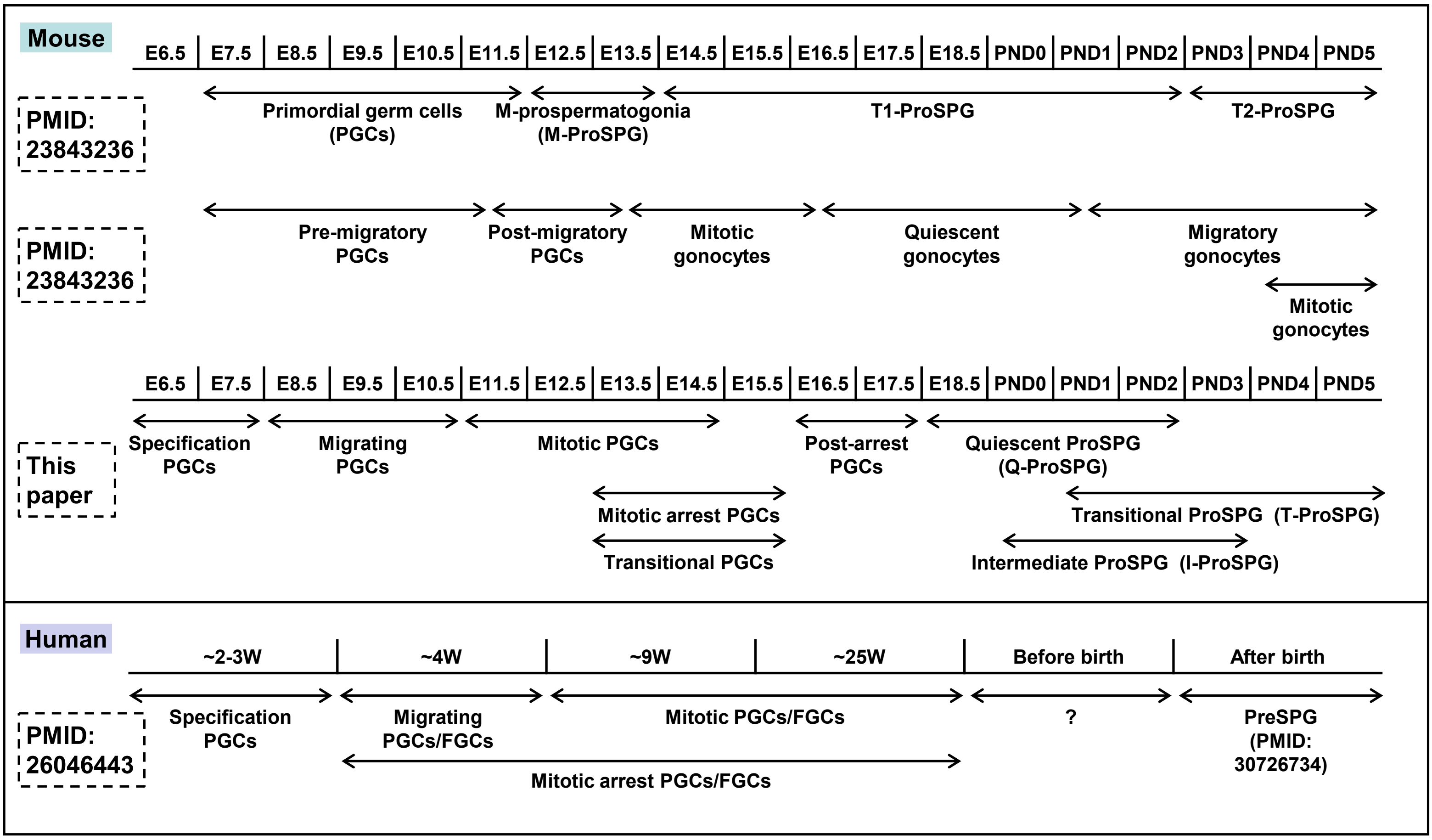

Supplementary Fig. 15 Schematic representation of the time-lines of germ cell development in mouse and human. Modified from available literatures: PMID: 23843236 and PMID: 19306346. M, mitotic; T1, transitional 1; T2, transitional 2.

Supplementary Fig. 16

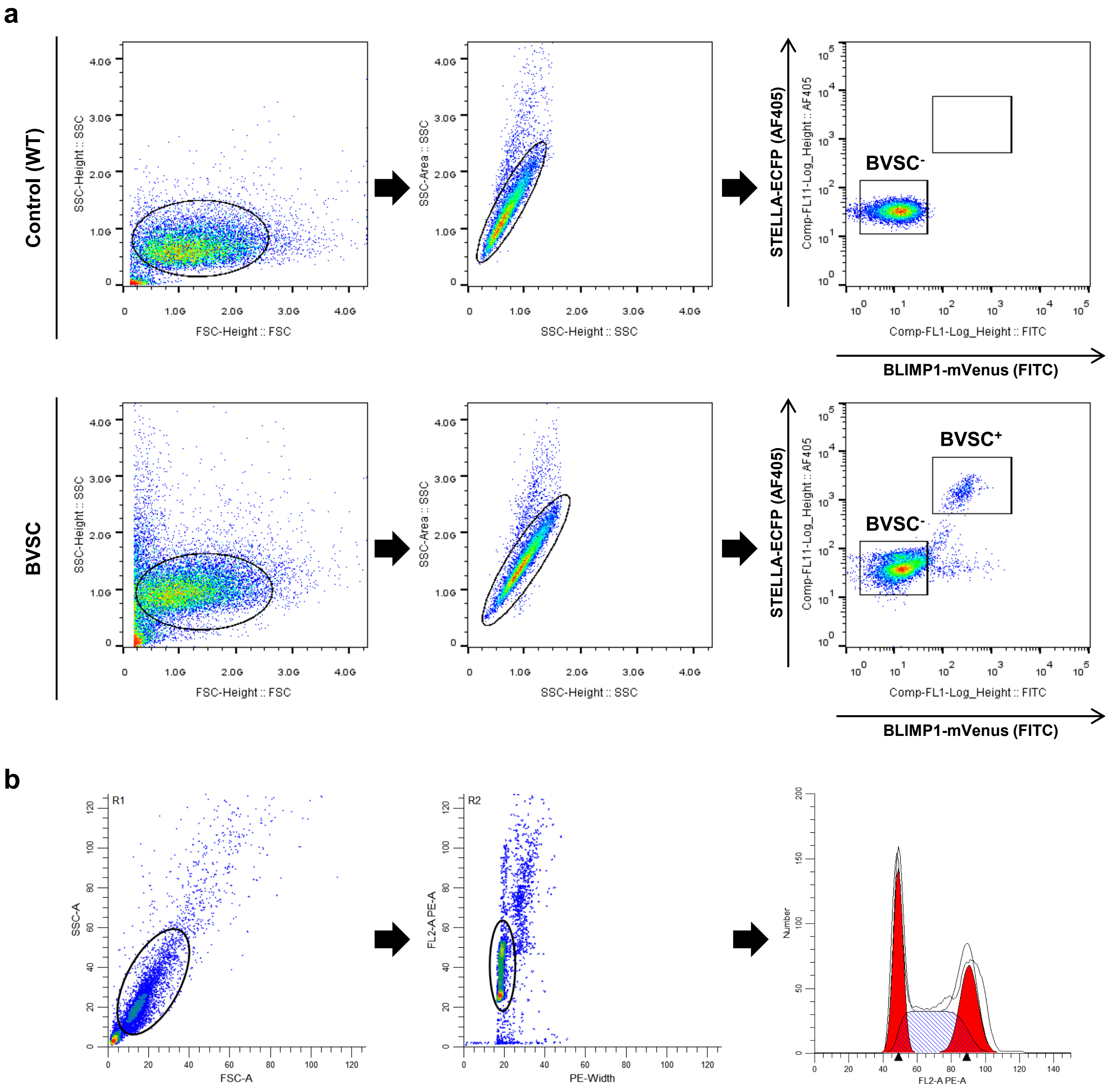

Supplementary Fig. 16 Cell sorting and cell cycle analysis by FACS. a, Representative FACS gating scheme for the sorting of prenatal germ cells and somatic cells (E16.5). Wild-type gonadal cells at the same time-point were served as gating control. Intact cells are identified on the basis of forward scatter (FSC-A) and side scatter (SSC-A) characteristics, and cell debris and aggregates were excluded. Germ cells ($BVSC^{+}$) and somatic cells ($BVSC^{-}$) were then identified based on the expression of mVenus and ECFP. **b,** Representative scheme for the cell cycle analysis. Intact cells are identified on the basis of forward scatter (FSC-A), side scatter (SSC-A) and PE-A characteristics, and cell debris and aggregates were excluded. The DNA content was then analyzed by ModFit software.
