# Supplementary material for: Cell-fate transition and determination analysis of mouse male germ cells throughout development
Source: Nat Commun. 2021 Nov 25;12:6839. doi: 10.1038/s41467-021-27172-0 (PMC8617176; doi:10.1038/s41467-021-27172-0)
Supplement: Supplementary file 3 — Description of Additional Supplementary Files [file 41467_2021_27172_MOESM3_ESM.docx]

Description of Additional Supplementary Files

File Name: Supplementary Data 1

Description: Sample information of 11,598 germ cells and testicular somatic cells from male mice, 43 G1/S and 54 G2/M cell-cycle-related genes, global DEGs among 18 germ cell clusters, and genes related to pluripotency used in SOM plot.

File Name: Supplementary Data 2

Description: DEGs of epiblast cells, mix mesoderm cells, and specification PGCs, DEGs and GO terms of three states of migrating PGCs, DEGs and GO terms of mitotic PGCs, transitional PGCs, mitotic arrest PGCs, post-arrest PGCs, Q-ProSPG, and T-ProSPG, DEGs of mitotic PGCs, transitional PGCs, and mitotic arrest PGCs, DEGs and GO terms of transitional PGCs with mitotic PGCs and mitotic arrest PGCs, and list and GO Terms of mitotic to mitotic arrest transition-related genes.

File Name: Supplementary Data 3

Description: List and GO Terms of 156 cell cycle-related genes associated with male infertility or testicular cancer, sample information of 1,094 germ cells and testicular somatic cells from *Helq^-/-^* male mice, and DEGs and GO terms of mitotic PGCs, transitional PGCs, mitotic arrest PGCs, and E18.5 ProSPG in two genotypes, and misregulated mitotic to mitotic arrest transition-related genes from Fig. 3e.

File Name: Supplementary Data 4

Description: DEGs and GO terms of mitotic arrest PGCs, Q-ProSPG, T-ProSPG, undiff.ed SPG, diff.ing SPG, diff.ed SPG, DEGs and GO terms of two consecutive stages between mitotic arrest PGCs and undiff.ed SPG, and GO analysis of each spermatogenic cell stage with their former- and later-stage cells

File Name: Supplementary Data 5

Description: Sample information, DEGs, and GO terms of sh-*Hhex* and sh-NC mSSCs.

File Name: Supplementary Data 6

Description: DEGs and SCENIC results of different cell types in human and mouse in PGCs and SPG, mutant phenotypes of representative DEGs in mouse reproductive system from MGI database, and list of the intersection between mouse mitotic to mitotic arrest transition-related genes (Fig. 3e) and male infertility-, nonobstructive azoospermia-,and testicular germ cell tumor-related genes.

File Name: Supplementary Data 7

Description: Materials and software used.
